# Outcomes of the Tree Theme Method versus regular occupational therapy: A longitudinal follow‐up

**DOI:** 10.1111/1440-1630.12796

**Published:** 2022-03-07

**Authors:** A. Birgitta Gunnarsson, Carita Håkansson, Katarina Hedin, Petra Wagman

**Affiliations:** ^1^ Department of Health and Rehabilitation, Institute of Neuroscience and Physiology University of Gothenburg Gothenburg Sweden; ^2^ Department of Research and Development, Region Kronoberg Växjö Sweden; ^3^ Division of Occupational and Environmental Medicine Lund University Lund Sweden; ^4^ Futurum, Region Jönköping County and Department of Health, Medicine and Caring Sciences Linköping University Linköping Sweden; ^5^ Department of Clinical Sciences in Malmö, Family Medicine Lund University Lund Sweden; ^6^ Department of Rehabilitation, School of Health and Welfare Jönköping University Jönköping Sweden

**Keywords:** anxiety, creative activities, depression, intervention, randomised controlled trial

## Abstract

**Introduction:**

Depression and anxiety disorders affect individuals' everyday lives, and treatments that can help them to perform everyday occupations are needed. Occupational therapy for this group has been evaluated from a short‐term perspective but not from a long‐term perspective; further research is thus warranted. The aim of the study was to investigate the longitudinal outcomes of the Tree Theme Method (TTM) compared with care as usual, provided by occupational therapists, in terms of everyday occupations, psychological symptoms, and health‐related aspects.

**Methods:**

This randomised controlled trial comprised a follow‐up 3 and 12 months after an intervention. A total of 118 participants (19–64 years) with depression or anxiety disorders and problems with everyday occupations completed the base line questionnaires, 100 completed the follow‐up at 3 months, and 84 completed the follow‐up at 12 months. Imputations of missing data were performed using the last observation, and parametric analysis was used.

**Results:**

Both groups showed significant improvements (*P* value ≤ 0.01) in everyday occupations, psychological symptoms and health‐related aspects after 3 and 12 months. No significant differences were found between the groups.

**Conclusion:**

This study contributes with knowledge about the outcomes of occupational therapy for clients living with depression and anxiety disorders. Both TTM and care as usual lead to significant improvements over time concerning everyday occupations, psychological symptoms, and health‐related aspects. The fact that both occupational therapy methods were associated with improvements for clients with depression and anxiety supports client‐centredness in enabling an occupational therapist to choose the method best suited for the individual.

**Key Points for Occupational Therapy**

- Both TTM and regular occupational therapy were associated with significant, longitudinal improvements in everyday occupations, psychological symptoms and health‐related aspects.
- Occupational therapy could be clinically useful for clients with depression and anxiety.
- Several occupational therapy interventions for clients living with depression and anxiety can be used.

## 1 INTRODUCTION

Depression and anxiety disorders are increasing globally (Abrams et al., 2020), not least in Sweden (Försäkringskassan, 2020), and also to a greater extent among women than men (Abrams et al., 2020; Lamers et al., 2011). Depression entails symptoms of reduced mood and diminished interest and pleasure in activities and an anxiety disorder entails symptoms such as being worried about various events and activities in everyday life (American Psychiatric Association, 2013). Both these disorders involve difficulties in performing everyday occupations for the individuals and their families and also lead to significant costs due to sickness absence and loss of productivity in society (Sobocki et al., 2007). Medical treatment and psychological interventions are usually recommended (NICE, 2009, 2011), but there are many people who do not get the treatment and support they need or the interventions that are received are not sufficient (WHO, 2021). The treatment strategies provide focus primarily on reducing symptoms of depression and anxiety. However, there is insufficient knowledge concerning the relationship between reduced symptoms and increased ability to perform everyday occupations. Treatment methods focusing on occupational performance for persons living with depression and anxiety are thus needed.

A number of occupational therapy interventions for clients with mental illnesses were presented in a recent review (Kirsh et al., 2019). These showed that creative activities and activity‐based interventions could be useful for enhancing the ability to perform everyday occupations. However, the focus in a majority of the research has been clients who have performed creative activities and/or activity‐based methods and who lived with severe mental illnesses, such as schizophrenia (Kirsh et al., 2019). Some studies have included people with depression and anxiety disorders. For example, in a study by Yamashita et al. (2012), the clients performed creative activities, such as art therapy, crafts, and gardening. The intervention demonstrated the effects on the clients' psychiatric symptoms directly after each session, although no effects were found after the clients had completed 25 treatment sessions (Yamashita et al., 2012). The Tree Theme Method (TTM) (Gunnarsson et al., 2006) was also referred to in the review (Kirsh et al., 2019). The TTM is a client‐centred occupational therapy intervention based on the theories of creative activities, occupational storytelling and meaningful occupations (Gunnarsson et al., 2006). It has been developed for clients living with depression and anxiety disorders. The outcomes, measured at baseline and directly after the completed intervention, revealed significant improvements concerning everyday occupations, psychological symptoms and health‐related aspects in a pre‐test post‐test study without any control group (Gunnarsson & Eklund, 2009). Furthermore, comparing TTM and regular occupational therapy in a randomised controlled trial revealed significant improvements in both groups directly after the intervention but not between interventions (Gunnarsson et al., 2018).

In a majority of the research conducted on clients with depression and anxiety disorders, they have solely been assessed prior to the intervention and directly after it, that is, with a short‐term perspective (Knekt et al., 2011). A long‐term perspective can entail two different approaches: either a longer period of treatment, for example, as performed in the study by Yamashita et al. (2012) or a follow‐up of outcomes at a certain time after treatment completion. The latter was performed in a 3‐year follow up of the TTM without a control group (Gunnarsson & Björklund, 2013) and showed positive outcomes in terms of everyday occupations, psychological symptoms and health‐related aspects. We aim to investigate whether these positive outcomes could be replicated or not in the present study, as well whether the outcomes related to the TTM differ from care as usual provided by occupational therapists. Otherwise, we have not identified any studies of occupational therapy interventions based on creative activities for people with depression and anxiety that have evaluated the effects several months after completed intervention. It is therefore also valuable to evaluate the long‐term outcomes of the intervention.

The aim of this study was thus to investigate the longitudinal outcomes of the TTM intervention compared with care as usual provided by occupational therapists in terms of everyday occupations, psychological symptoms, and health‐related aspects, 3 and 12 months after an intervention for people living with depression and/or anxiety disorders.

## 2 METHODS

### 2.1 Ethical considerations

This study was approved by the Regional Ethical Review Board in Linköping, Sweden (no. 2012/232‐31 and no. 2015/12‐3220). The ethical principles of the Declaration of Helsinki concerning information, voluntariness, the possibility to withdraw at any time, and confidentiality were followed. Written informed consent was obtained from all participants.

### 2.2 Design

This longitudinal study involved a 3‐ and 12‐month follow‐up in a randomised controlled trial (RCT). The study was registered as a Clinical Trial NCT01980381 and has previously been described in a study protocol (Gunnarsson et al., 2015). Data on baseline and follow‐up 1 to 2 weeks directly after treatment have also previously been published (Gunnarsson et al., 2018).

### 2.3 Participants and context

This RCT included participants aged 18–65 years, diagnosed with depression (ICD‐10 = F30‐F39) and/or anxiety disorders (ICD‐10 = F40‐F48) (WHO, 2020) and assessed by an occupational therapist as having difficulties performing everyday occupations.

The study was conducted in primary health care centres and general outpatient psychiatric care units in three counties in Southern Sweden. Occupational therapists, specifically trained in the TTM, recruited and performed the allocated interventions with the participants.

### 2.4 Interventions

#### 2.4.1 Tree Theme Method (TTM)

The TTM (Gunnarsson et al., 2006) comprised five sessions. The method involved the participants painting pictures of trees representing their present life situation, their childhood, adolescence, and adulthood and using these as starting points for a dialogue with an occupational therapist in which they told their occupational life story. The pictures of each tree and the occupational storytelling mirrors the individual, their activities and interests, and relationships with others. By reflecting about earlier periods of their lives, including strengths as well as limitations in everyday life, the individual may use, or change an activity or interest in their present life. During the fifth and final session, all their previous pictures of trees and the dialogue are used as a starting point for painting a picture of their future tree in order to reflect and develop strategies to cope with their future everyday occupations. At the end of each session, the individual decides about a personal task, which is related to their problems and needs in everyday life and that task is to be completed prior to the next session.

#### 2.4.2 Care as usual provided by occupational therapists

The care as usual provided by occupational therapists comprised five sessions. All participating occupational therapists documented what they meant with care as usual. The sessions varied depending on occupational therapist and were mainly focussed on dialogues and activities concerning daily rhythm, daily routines, and everyday occupations. A session could, for example, involve planning and scheduling activities at home and at work, prescribing aids to handle symptoms of anxiety and achieve better sleep, or to visit the individual's workplace. The participants also had a possibility to decide about a personal task related to their problems and needs in everyday life, which was to be completed prior to the next session.

The same occupational therapist performed both the TTM intervention and the care as usual because it had been found in a previous study related to the TTM (Gunnarsson & Eklund, 2009) that the therapeutic relationship was associated with positive outcomes. Two different fidelity forms were completed: one for the TTM and one for care as usual. The fidelity forms for the TTM concerned the use of progressive relaxation, painting pictures representing various periods of everyday life, occupational storytelling, and home tasks. The fidelity forms for care as usual concerned the same aspects as for the TTM but not the use of progressive relaxation, painting pictures of trees, or occupational storytelling.

### 2.5 Procedure for data collection

Potential participants for the study were first identified by health service occupational therapists. Those who were willing to participate then met a project assistant, who was responsible for the data collection. Baseline data were collected after receiving informed consent. The data included gender, age, living status, having children, education level, primary support, use of medication, primary diagnoses, and treatment received due to mental illness in the last month. The questionnaires were then completed, and thereafter, the participant was allocated to TTM or care as usual. The participants were contacted by their project assistant via phone throughout the project, who also asked them to take part in the follow‐up. The project assistants were unaware of which treatment each participant had been allocated to. The recruitment of the first participant started in 2013, and the last participant completed the 12‐month follow up in 2017.

### 2.6 Primary outcomes

Primary outcomes were occupations (performance, satisfaction with everyday occupations, and occupational balance) and psychological symptoms of anxiety and depression.

#### 2.6.1 Measurements

*The Canadian Occupational Performance Measurement* (COPM) (Law et al., 1998) is a semi‐structured interview in which the participant identifies difficulties with specific tasks in self‐care, unpaid/paid productivity, and leisure. Participants rate their performance and satisfaction with the performance of these specific tasks on a 10‐step scale (1–10); the higher the score, the higher the level of performance and satisfaction with the performance, despite difficulties with the identified tasks. Changes over time with two or more steps are considered to be clinically significant (Law et al., 1998). The Swedish version of the COPM has shown good internal, consistency (Gunnarsson et al., 2018), high responsiveness to change (Wressle et al., 1999), and usefulness in clinical practice (Wressle et al., 2002).

*The Satisfaction with Daily Occupations* (SDO) (Eklund, 2004) scale is a semi‐structured interview, focusing on whether or not the participant currently performs various occupations (“yes”/”no”) in self‐care, domestic tasks, work, and leisure. Participants rate their satisfaction with the specific performance/non‐performance on a seven‐step scale (1–7); the higher the score, the higher the level of satisfaction with their performance/non‐performance. The measurement has shown good internal consistency, reliability (Eklund & Gunnarsson, 2007), and validity (Eklund & Gunnarsson, 2008).

*The Occupational Balance Questionnaire* (OBQ) (Wagman & Håkansson, 2014) is a questionnaire comprising 13 items in which participants rate their perceptions of balance in everyday occupations on a six‐step scale (0–5); the higher the score, the higher the occupational balance. The measurement has shown good internal consistency in the present sample (Gunnarsson et al., 2018) and test–retest reliability (Wagman & Håkansson, 2014).

*The Symptom Checklist‐90‐R* (SCL‐90‐R) (Derogatis, 1992) is a questionnaire comprising 90 items in which participants rate their psychological problems on a five‐step scale (0–4); the higher the score, the higher the level of severity. The SCL‐90‐R consists of nine symptom dimensions: depression, anxiety, phobic anxiety, hostility, obsessive–compulsive, interpersonal sensitivity, somatization, paranoid ideation and psychoticism, and three global indexes: the Personality Symptom Index, the Positive Symptom Distress Index (PSDI), and the Global Severity Index, in which the main global index of distress is the latter. We used the depression and anxiety scales and the Global Symptom Index (GSI) from the SCL‐90‐R in this study. The Swedish version has shown good internal consistency (Gunnarsson et al., 2018), reliability, and validity (Fridell et al., 2002).

*The Hospital Anxiety and Depression Scale* (HADS) (Zigmond & Snaith, 1983) is a questionnaire comprising 14 items in which participants rate their symptoms of anxiety and depression on a four‐step scale (0–3); the higher the score, the higher the level of severity. The HADS is divided into two subscales, measuring anxiety (seven items) and depression (seven items). Scores ≤6 indicated no state of anxiety or depression, scores from 7 to 10 indicated possible anxiety or depression, and scores ≥11 indicated probable severe anxiety or depression. The measurement has shown good internal consistency (Gunnarsson et al., 2018), good construct validity, and usefulness in assessing symptoms of anxiety and depression (Bjelland et al., 2002).

*The Montgomery‐Åsberg Depression Rating Scale* (MADRS‐S) (Montgomery & Åsberg, 1979) is a questionnaire comprising nine items in which participants rate their level of symptoms of depression on a seven‐step scale (0–6); the higher the score, the higher the severity. Scores ≤12 were classified as no depression, scores from 12 to 19 were classified as mild depression, scores from 20 to 34 were classified as moderate depression, and scores ≥35 were classified as severe depression. The measurement has shown good internal consistency (Gunnarsson et al., 2018) and usefulness as a screening instrument (Svanborg & Åsberg, 2001).

### 2.7 Secondary outcomes

Secondary outcomes were various health‐related aspects, including sense of coherence, sense of control in everyday life and quality of life.

#### 2.7.1 Measurements

*The Sense of Coherence Scale* (SOC) (Antonovsky, 1993) is a questionnaire comprising 13 items in which participants rate how well they experience life as comprehensive, manageable, and meaningful on a seven‐step scale (1–7); the higher the score, the higher the sense of coherence. The measurement has shown good internal consistency in the present sample (Gunnarsson et al., 2018) and has been related to good mental health (Eriksson & Lindström, 2005).

*The Mastery Scale* (Marshall & Lang, 1990) is a questionnaire comprising seven items in which participants rate their sense of control in everyday life on a four‐step scale (1–4); the higher the score, the higher the sense of control. The measurement has shown good internal consistency (Eklund et al., 2012; Gunnarsson et al., 2018) and validity (Eklund et al., 2012).

*The Manchester Short Assessment of Quality of Life* (MANSA) (Priebe et al., 1999) is a questionnaire comprising 12 items in which participants rate their general life satisfaction, and satisfaction with work, economic situation, relationships to others, leisure, housing situation, physical and mental health on a seven‐step scale (1–7); the higher the score, the higher the level of satisfaction. The measurement has shown good internal consistency (Gunnarsson et al., 2018), validity and reliability (Björkman & Svensson, 2005).

### 2.8 Sample size, randomization, and allocation

**FIGURE 1:**
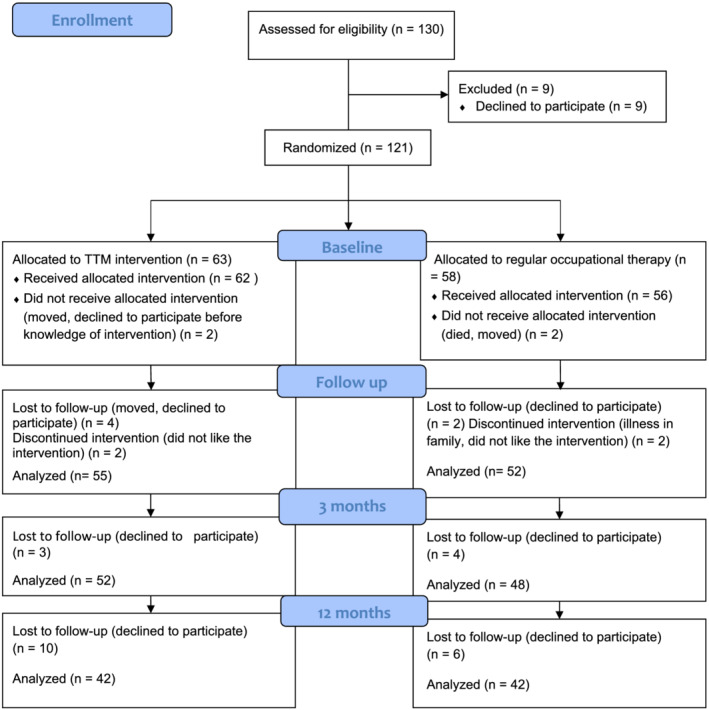
Flow diagram of the study progress

The power calculation indicated a need of 120 participants in order to attain 80 percentage power (*p* = 0.05). An administrator performed a blocked randomization. The participants did not know their individual allocation prior to giving informed consent and completing the baseline data collection. There were 118 participants who completed the baseline data questionnaire; 100 participants completed the follow up at 3 months; and 84 participants completed data at follow up at 12 months after the intervention (Figure 1).

### 2.9 Data analysis

Data quality was verified by a project assistant and the first author. Imputations of missing data were performed by carrying forward the last observation (Salkind, 2010). Data were approximately normally distributed. Repeated measures ANOVA was used to analyse changes over time, comparing the outcomes between groups as a fixed factor, and time as a random factor. The IBM SPSS Statistics 27.0 was used for the analyses.

## 3 RESULTS

There were 10 men and 52 women (mean age 43.0 years, SD 11.3, range 19–63 years) in the TTM intervention group and 10 men and 46 women (mean age 40.1 years, SD 12.6, range 19–64 years) in the care as usual group at baseline. No significant differences were found concerning participant characteristics between the TTM intervention group and the care as usual group at baseline (Gunnarsson et al., 2018). A few participants dropped out of the allocated treatment, whereas most of them received their allocated treatment and also chose to complete the follow‐ups (Figure 1). There were no statistically significant differences in any baseline characteristics between those who did complete all follow‐ups and those who did not. Furthermore, we found no statistically significant differences in outcomes between those who received their treatment at primary health care centres and those who received their treatment in general outpatient psychiatric care units. Similarly, no statistically significant differences in baseline characteristics were found between those participants who had improved or who had not improved, in terms of everyday occupations, psychological symptoms, and health‐related aspects regarding participant characteristics, severity of symptoms of depression and anxiety, and sense of coherence (Table 1).

**TABLE 1:** Comparison of baseline characteristics for the participants diagnosed with depression or anxiety disorders

	Baseline (N = 118)		
	TTM (n = 62)n (%)	Care as usual(n = 56) n (%)	*P* value(chi‐squared test)
Gender			
Women	52 (83.9)	46 (82.1)	0.80
Primary diagnosis			0.44
Affective disorders (F31‐38)	40 (64.5)	40 (71.4)	
Anxiety/obsessive disorders (F40‐49)	22 (35.5)	16 (28.6)	
Living status			
With someone	43 (69.4)	32 (57.1)	0.37
Have children ≤18 years	23 (37.1)	18 (32.1)	0.77
Education			0.63
University	20 (32.3)	15 (26.8)	
High school degree	31 (50.0)	27 (48.2)	
Elementary school	9 (14.5)	13 (23.2)	
No elementary school	2 (3.2)	1 (1.8)	
Employment			0.62
Employed/student	23 (37.1)	16 (28.6)	
Unemployed	4 (6.5)	6 (10.7)	
Others (parental leave, retired)	1 (1.6)	1 (1.8)	
Sick‐leave (including 3 who work trained)	34 (54.8)	33 (58.9)	
Medications			
Sedatives	25 (40.3)	29 (51.8)	0.32
Antidepressants	43 (69.3)	41 (73.2)	0.45
Anxiolytics	19 (30.6)	23 (41.1)	0.48
Others (e.g., antipsychotics, central stimulants)	12 (19.4)	10 (17.9)	0.84

### 3.1 Everyday occupations

Both the TTM intervention group and the care as usual group improved their performance of everyday occupations significantly, as well as their satisfaction with this from baseline to 3 months after completion of the treatment, and to a greater extent 12 months after its completion. Both groups improved the COPM, but there were no significantly differences between groups (Table 2). Both the TTM intervention group and the care as usual group improved significantly on the SDO activity level and the longitudinal satisfaction scale. Both the TTM intervention group and the care as usual group scored a statistically significant higher occupational balance (OBQ) 3 months and 12 months after completion of the treatment. There were no significant, longitudinal differences in any of the everyday occupation variables between the groups (Table 2).

**TABLE 2:** Levels of the primary and secondary outcomes in the TTM group and the care as usual group at baseline, 3 months, and 12 months after intervention

	The TTM group (n = 62)			Care as usual (n = 56)			P values	
	Baseline mean (SD)	3 months after completed intervention mean (SD)	12 months after completed intervention mean (SD)	Baseline mean (SD)	3 months after completed intervention mean (SD)	12 months after completed intervention mean (SD)	Between time points^a^	Between treatments^b^
Primary outcomes								
COPM								
Performance	3.86 (1.52)	4.70 (2.06)	5.21 (2.13)	3.63 (1.45)	4.73 (1.99)	5.47 (1.91)	≤0.01	0.94
Satisfaction	2.71 (1.35)	4.26 (2.34)	4.75 (2.51)	3.32 (1.74)	4.50 (2.29)	5.14 (2.42)	≤0.01	0.23
SDO								
Activity level	7.23 (2.22)	7.16 (2.47)	7.65 (2.49)	7.45 (2.28)	8.05 (2.00)	8.20 (2.28)	0.01	0.13
Satisfaction score	61.02 (16.52)	66.30 (17.96)	69.57 (17.52)	57.89 (17.23)	62.86 (13.48)	67.02 (16.88)	≤0.01	0.25
OBQ	22.87 (10.03)	28.27 (13.24)	30.44 (13.96)	22.96 (9.79)	28.32 (10.57)	32.70 (12.77)	≤0.01	0.68
SCL‐90‐R symptom scales								
Depression	82.69 (18.46)	75.32 (23.03)	73.42 (23.64)	81.66 (18.04)	73.93 (18.72)	71.55 (20.60)	≤0.01	0.68
Anxiety	83.00 (23.04)	77.55 (27.80)	75.56 (27.95)	84.57 (22.84)	77.09 (22.90)	75.34 (25.29)	≤0.01	0.95
SCL‐90‐R indexes								
GSI	84.65 (22.36)	78.35 (28.30)	76.74 (28.69)	82.27 (20.37)	73.79 (18.84)	71.73 (19.54)	≤0.01	0.33
Positive symptoms	74.60 (13.54)	67.89 (17.42)	66.87 (17.69)	70.30 (12.04)	64.82 (13.33)	62.87 (13.34)	≤0.01	0.13
Positive symptoms total	70.19 (11.40)	66.90 (14.57)	65.53 (15.38)	70.96 (10.80)	66.79 (11.19)	65.29 (12.78)	≤0.01	0.95
MADRS‐S	25.03 (8.89)	20.65 (11.11)	19.42 (11.39)	25.16 (8.37)	20.98 (9.32)	17.45 (9.76)	≤0.01	0.76
HADS	23.74 (7.63)	19.95 (9.37)	18.05 (9.71)	23.38 (7.53)	20.01 (7.50)	17.91 (7.75)	≤0.01	0.92
HADS‐A	13.68 (4.27)	11.58 (5.11)	10.79 (5.30)	13.39 (4.24)	11.77 (4.41)	10.91 (5.00)	≤0.01	0.94
HADS‐D	10.06 (4.55)	8.37 (5.11)	7.26 (5.06)	10.07 (4.07)	8.19 (4.57)	6.62 (3.92)	≤0.01	0.64
Secondary outcomes								
SOC	47.10 (12.64)	51.40 (16.18)	54.06816.99)	49.00 (11.38)	52.48 (11.74)	54.87 (13.45)	≤0.01	0.59
Mastery	17.82 (3.57)	18.35 (4.73)	19.23 (4.88)	17.18 (3.12)	18.04 (3.68)	18.57 (3.94)	≤0.01	0.41
MANSA	46.52 (11.28)	49.08 (15.35)	51.23 (15.79)	44.11 (11.35)	47.48 (11.90)	50.59 (14.72)	≤0.01	0.49

Abbreviations: COPM, Canadian Occupational Performance Measure; GSI, Global Symptom Index; HADS, Hospital Anxiety and Depression Scale divided into an Anxiety subscale (HADS‐A) and a Depression subscale (HADS‐D); MADRS‐S, Montgomery‐Åsberg Depression Rating Scale; MANSA, Manchester Short Assessment of quality of life; OBQ, Occupational Balance Questionnaire; SCL‐90‐R, Symptom Checklist‐90‐R; SDO, Satisfaction with Daily Occupations; SOC, Sense of Coherence measure.

^a^ Between time points = the systematic differences between the time points, for a given treatment, that is, TTM and Care as usual respectively.

^b^ Between treatments = the systematic difference between treatments TTM and Care as usual at each time point.

### 3.2 Psychological symptoms

The TTM intervention group and the care as usual group reduced their symptoms of depression and anxiety significantly from baseline to 3 months after completion of treatment, and a further reduction was seen 12 months after completion of the treatment. There were no significant, longitudinal differences in psychological symptoms between the groups (Table 2).

### 3.3 Health‐related aspects

There were significant, longitudinal improvements for all ratings of health‐related aspects in both the TTM intervention group and in the care as usual group. There were no significant, longitudinal differences in health‐related aspects between the groups (Table 2).

## 4 DISCUSSION

The results of the present study add knowledge about the long‐term outcomes of the two types of occupational therapy interventions. This knowledge is valuable because not only is there a lack of knowledge about interventions based on creative activities and activity‐based methods in a long‐term perspective (Kirsh et al., 2019), but also a lack of knowledge about occupational therapy interventions in general. Furthermore, most research in this field focuses on clients with severe mental illness, and not clients living with depression and anxiety disorders (Gutman & Brown, 2018). Our results in terms of the significant improvements in everyday occupations and health‐related aspects in both groups, thus supplement the previous results relating to improvements directly after treatment completion (Gunnarsson et al., 2018), as well as the follow‐up without any control group (Gunnarsson & Björklund, 2013). Moreover, the results from this study show even further improvements 3 and 12 months after treatment completion. These outcomes are in contrast with the findings in the study by Yamashita et al. (2012). These authors showed positive outcomes directly after each session of creative activities for people living with depression, but not after completing the long‐term treatment procedure of 25 sessions. The differences between the TTM and the study performed by Yamashita et al. (2012) could be due to how creative activities are used as a tool in occupational therapy. The TTM is a method that involves painting pictures of trees as a starting point to reflect and develop strategies in order to promote changes in the client's everyday occupations. The clients in the study by Yamashita et al. (2012) received art therapy, crafts, and gardening as activities in an occupational therapy context, but without explicit focus on necessary changes in everyday life.

Both interventions in the present study revealed significant improvements in everyday occupations, such as changes in occupational performance. The significant improvements meant that both groups improved their ratings on the COPM by almost two steps, which according to Law et al. (1998) amounts to a clinical utility level. Both groups also rated improvements in satisfaction with occupational performance, measured by the COPM (Law et al., 1998) and the SDO (Eklund, 2004). This may suggest that occupational therapy not only contributes to enhancing the ability to perform everyday occupations but can also provide an intervention to reduce psychological symptoms.

Furthermore, the present outcomes are similar to those in another RCT, in which adjuvant occupational therapy was compared with care as usual (Hees et al., 2013) in a short‐term and a long‐term perspective (18 months). The clients in the latter study were employees on sick leave who lived with depression. The focus was on work ability, psychological symptoms and health‐related aspects. Improvements were reported for both the intervention and control groups in both the latter study and the present one, whereas no significant differences were found. This can be considered to be a limitation but may also be seen as representing a clinical strength, implying that care as usual provided by occupational therapists can be useful as a complement to other treatments for clients living with depression and anxiety disorders.

Moreover, different types of interventions are required, as no single method suits everyone, and the “wrong” method for a person could lead to drop‐outs and clients who do not complete their treatment (Ekeblad et al., 2016). The participants in the present RCT appear to have benefitted from both the TTM intervention and care as usual. This may also highlight the importance of interventions being client‐centred (Hamovitch et al., 2018) so that the client and therapist develop a working relationship and in dialogue identify goals and the intervention that works best for each client (Flückiger et al., 2018).

### 4.1 Methodological considerations/limitations

This study has limitations as well as strengths. The internal validity (Kazdin, 2021) was strengthened by both types of occupational therapy interventions focusing on everyday occupations aimed at enhancing the participant's ability to cope with everyday life. The reliability was strengthened by the project assistants, who were all occupational therapists, receiving training in the instruments and data collection to ensure that the latter would be performed in as similar a way as possible for each participant. It can be difficult to avoid attrition in a study with a long‐term perspective, and it should be recognised that this RCT had fewer participants for the follow‐ups at 3 and 12 months. However, the power calculation was made for the main outcomes (after treatment) (Gunnarsson et al., 2018). The principle of intention‐to‐treat by imputations of missing data was thus used to reduce the risk for bias due to attrition.

A longitudinal study can contain limitations in terms of history (Kazdin, 2021). In order to address this, the project assistants in the present study asked the participants at the time of follow‐up whether any specific events had occurred since the previous data collection. Some specific events were reported by the participants, but these were similar in both groups. Another history limitation is that we did not know anything about disease duration, that is, years since the participants received their diagnosis. A further limitation was that there was some participant attrition, mostly at the later follow‐ups, although there were no statistically significant differences in characteristics between those who did complete all follow‐ups and those who did not. It can, however, also be seen as a strength that 75% of the participants chose to complete all data collections.

A limitation and a potential bias could be that the same occupational therapist, trained in the TTM, performed both interventions. The occupational therapists in this project were requested to define what they meant with five sessions of care as usual. This, however, showed to be a more focussed therapy than they usually provided, which may partly be due to specifying the five sessions. This fact highlights a limitation, which may have been avoided if we had added a control group who did not receive any occupational therapy at all, which not was possible due to ethical reasons when the participants were in need of occupational therapy A further limitation was that we did not choose to validate the two different occupational therapy treatments against other forms of treatment. Different results would potentially have been identified if the study had also included a third group who received cognitive behavioural therapy, an evidence‐based method, commonly used and recommended (Socialstyrelsen, 2017) but which does not focus on everyday occupations. Future research is thus warranted concerning the effects of the TTM in terms of psychological symptoms, health‐related aspects and everyday occupations, including interventions other than occupational therapy.

The RCT‐design used in the present study strengthened the external validity (Kazdin, 2021). The purposeful sampling and the fact that the two groups were comparable in terms of basic characteristics (Gunnarsson et al., 2018) can be said to further strengthen the external validity. These findings could thus be generalised to other groups of clients living with depression and anxiety disorders. More women than men participated, thus preventing the generalisation of the results of the present study to men. The gender distribution was more skewed than desirable in the present study, even though it has been shown that depression and anxiety disorders are more common in women (Abrams et al., 2020; Lamers et al., 2011).

## 5 CONCLUSION

This study showed that both the TTM and care as usual provided by occupational therapists were associated with positive improvements in a long‐term perspective concerning everyday occupations, psychological symptoms and health‐related aspects. This suggests that these occupational therapy methods could be clinically useful and serve as a complement to the standard treatment repertoire, for example, medical treatment and psychological interventions, for clients living with depression and anxiety disorders. Occupational therapists thus need to have a client‐centred approach and to choose the best occupational therapy intervention, depending on the client's needs.

## CONFLICT OF INTEREST

The authors have no conflict of interest to declare.

## AUTHOR CONTRIBUTIONS

A. B. G. conceived the study, formulated the research design and was responsible for the research process, obtained the funding, and was the primary author of the manuscript. C. H., K. H., and P. W. contributed to the study design and data analysis and manuscript writing. All authors read and approved the final manuscript.

## Data Availability

The dataset used and/or analyzed during the present study is available from the corresponding author on request.
